# Non-linear quantum-classical scheme to simulate non-equilibrium strongly correlated fermionic many-body dynamics

**DOI:** 10.1038/srep32940

**Published:** 2016-09-09

**Authors:** J. M. Kreula, S. R. Clark, D. Jaksch

**Affiliations:** 1 Clarendon Laboratory, University of Oxford, Parks Road, Oxford OX1 3PU, United Kingdom; 2 Department of Physics, University of Bath, Claverton Down, Bath BA2 7AY, United Kingdom; 3 Max Planck Institute for the Structure and Dynamics of Matter, Hamburg, Germany; 4 Centre for Quantum Technologies, National University of Singapore, 3 Science Drive 2, Singapore 117543, Singapore

## Abstract

We propose a non-linear, hybrid quantum-classical scheme for simulating non-equilibrium dynamics of strongly correlated fermions described by the Hubbard model in a Bethe lattice in the thermodynamic limit. Our scheme implements non-equilibrium dynamical mean field theory (DMFT) and uses a digital quantum simulator to solve a quantum impurity problem whose parameters are iterated to self-consistency via a classically computed feedback loop where quantum gate errors can be partly accounted for. We analyse the performance of the scheme in an example case.

Next generation scalable quantum devices^1^,^2^ promise a step change in our ability to do computations. Direct quantum simulation^3^,^4^,^5^ using highly controllable quantum systems^6^,^7^,^8^ has already led to numerous insights into many-body quantum physics, despite limitations in the size of the simulated system.

Recently, quantum computer simulations of strongly correlated fermion models have been proposed^9^,^10^. We suggest a hybrid quantum-classical scheme to simulate non-equilibrium dynamics of the Hubbard model in a Bethe lattice directly in the thermodynamic limit. Our scheme implements the non-equilibrium extension of the well-established dynamical mean-field theory (DMFT) method (for extensive reviews of DMFT, see, e.g. refs 11 and 12). Instead of the traditional all-classical method, the proposed scheme uses a digital quantum simulator to efficiently solve the DMFT impurity problem, the parameters of which are iterated to self-consistency via a classically computed feedback loop. This setup promises an exponential speed-up over the best currently-known Hamiltonian-based classical algorithms. We show how quantum gate errors can be partly accounted for in the feedback loop, improving simulation results. The scheme also avoids the sign problem in classical quantum Monte Carlo methods and works for all interaction strengths, unlike classical methods based on perturbation theory. Presently, non-equilibrium DMFT is one of the most promising methods to study time-dependent phenomena in high-dimensional correlated lattice models, and could thus be of interest for current efforts to develop scalable quantum technologies^1^,^6^,^13^,^14^. Examples of applications of non-equilibrium DMFT include the dielectric breakdown of Mott insulators^15^, damping of Bloch oscillations^16^, and thermalization after parameter quenches^17^,^18^.

Further to this, driven strongly correlated quantum materials are now being extensively investigated experimentally. A large motivation for this is the possibility of manipulating correlated phases of matter with strong pulses of light, such as photodoping of Mott insulators^19^ or inducing superconductivity^20^. The underlying physical mechanisms are, however, still poorly understood. Even the dynamical behaviour of conceptually simple and commonly used quantum lattice models is yet not fully grasped. Solving these model systems could elucidate physical phenomena underlying currently unexplained experimental results. A standard example of this kind of idealised model for non-equilibrium problems is the time-dependent Hubbard Hamiltonian


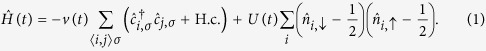


In this model, electrons with spin projections σ =↓,↑ move only between adjacent lattice sites *i* and *j* with timedependent ‘hopping’ energy *v*(*t*), where t denotes time. This process is described in the first sum, which is over all nearest-neighbour sites, with fermionic creation and annihilation operators 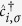 and 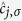, respectively. The electrons interact with Coulomb repulsion U(*t*) only if they occupy the same lattice site *i*, given in the latter term by the product of the number operators 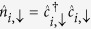 and 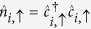.

This and similar models are extremely challenging to study numerically due to the exponential growth of the Hilbert space with system size. One thus often resorts to mean field approximations which typically consider only a single lattice site and replace interactions with its neighbourhood by a mean field Λ. This turns a linear quantum problem in an exponentially large Hilbert space into a much smaller but non-linear problem where Λ needs to be determined self-consistently. Such mean field approximations become increasingly accurate with the number of nearest neighbours. A classic example of this approach is the Weiss theory of ferromagnetism^21^. For mean field theory to be applicable to strongly correlated Fermi systems in thermal equilibrium, the mean field Λ_*σ*_(*t*) has to be dynamical to account for correlations between interactions with the environment that are separated by *t* in time, as schematically shown in Fig. 1a,b.

This highly successful approach is called DMFT^11^. DMFT can be extended to non-equilibrium systems^12^ by letting Λ_*σ*_(*t, t*′), which is often called hybridization function, depend on two interaction times *t* and *t*′ explicitly. Note that non-local spatial fluctuations can be included in DMFT by going beyond the single-site approximation and considering a cluster of isolated sites^22^,^23^, but this is beyond the scope of this work.

In general, it is a complex task to determine Λ_*σ*_(*t, t*′) and the related local single-particle Green’s function 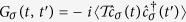 (where 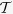 is the time-ordering operator), describing the response of the many-body system after a localized removal and addition of a particle at times *t* and *t*′. Commonly used numerical methods for solving the non-equilibrium DMFT problem include continuous-time quantum Monte Carlo, which suffers from a severe dynamical sign problem, and perturbation theory which can only address the weak and strong coupling regimes^12^.

In infinite dimensions, the system can also be explicitly mapped onto a single impurity Anderson model (SIAM)^24^


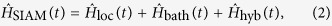



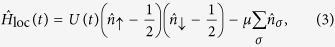



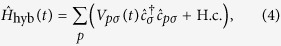



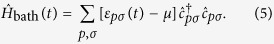


where the selected lattice site is represented by an impurity, with the creation (annihilation) operator 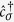 (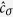) and number operator 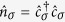, whose interaction with Λ_*σ*_(*t, t*′) is mimicked by a collection of *N* non-interacting bath sites with on-site energies *ε*_*pσ*_(*t*), as shown in Fig. 1c. The time-dependent hybridization energy *V*_*pσ*_(*t*) describes the amplitude for exchange of fermions between the impurity site and bath site *p*. These must be determined self-consistently: for given *V*_*pσ*_(*t*) the quantum dynamics of the SIAM is solved and its Green’s function and corresponding hybridization function Λ_*σ*_(*t, t*′) are determined. From Λ_*σ*_(*t, t*′) a new set of *V*_*pσ*_(*t*) is worked out which is then fed back into the SIAM. These steps are repeated until convergence is achieved^24^. The dynamics of the SIAM is usually worked out with exact diagonalization (ED)^24^ for small systems or with tensor network theory (TNT) methods^25^. However, the dynamical generation of entanglement in these problems has severely hampered the efficiency of TNT methods^25^,^26^. Furthermore, the required number of bath sites increases with the maximum simulation time *t*_max_. This makes solving the SIAM the exponentially difficult bottleneck^24^,^25^,^27^ in purely classical DMFT solvers.

Here, we propose and analyze a hybrid quantum-classical computing scheme for DMFT to efficiently solve the Hubbard model in a Bethe lattice. The Bethe lattice is chosen for the simplicity of its self-consistency condition. It is conceptually straightforward to extend the scheme to other types of lattices. A small digital quantum coprocessor solves the SIAM evolution with the resulting *G*_*σ*_(*t, t*′) being processed by a classical computer to complete the non-linear feedback loop as shown in Fig. 1d. We consider a trapped ion coprocessor for concreteness, although any other platform for quantum computing could implement the coprocessor as well. Even for imperfectly implemented quantum gates with realistic errors of 1% we find accurate solutions to a simple model problem in small systems. In addition, our numerical evidence suggests that gate errors mainly lead to a smearing of the bath energies, which can be accounted for in the classical feedback loop to improve the solution.

Figure 2 shows an example coprocessor quantum network for computing a contribution to the Green’s function (see Methods for details). The real and imaginary contributions to the impurity Green’s function are encoded as 〈*σ*^*z*^〉 and 〈*σ*^*y*^〉 of a probe qubit by interacting it with the impurity state at times *t*′ and *t* via controlled quantum gates^28^. We decompose the unitary dynamics 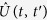 of the SIAM into a network of quantum gates^29^,^30^ by discretising time as *t*_*n*_ = *n*Δ*t*, where Δ*t* is a small time-step. We then breakup the evolution from *t* = 0 to *t* = *t*_*n*_ into a product of Trotter steps 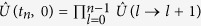. The Trotter steps can readily be implemented by single qubit rotations and multi-qubit entangling Mølmer-Sørensen (MS) gates^30^,^31^ that have recently been realized in ion traps with high fidelity^13^,^14^. The total number of MS gates per Trotter step scales only linearly with the number of bath sites.

We analyze the performance of our simulation scheme by considering a simple example system^24^. We study the infinite-dimensional time-dependent Hubbard model (1) with constant onsite interaction *U* and tunneling matrix element *v*(*t*). The simulation starts in the half-filled paramagnetic atomic limit with tunneling *v*(*t* = 0) = 0, which is then dynamically ramped up to its final value *v*_0_ after quench time 1/4*v*_0_ and is kept at *v*_0_ until the final simulation time *t*_max_ is reached^24^ (setting *ħ* = 1). Such a sudden quench is representative of experimental ultracold atom dynamics^32^,^33^ and also ultrafast dynamics probed in condensed matter systems^19^. The initial state of the system has a singly occupied impurity site in the completely mixed state of spin ↑ and spin ↓, and one half of the bath sites are doubly occupied and the other half empty (for explicit details, see ref. 24). In practice, we prepare the system in two pure fermion occupational number states, where one has the impurity in state |↑〉 and the other in state |↓〉, along with the bath states^24^. The results are then averaged over these two pure states. These initial number states are mapped onto product states of qubits via the Jordan-Wigner transformation (see Methods). The initial qubit configuration is that shown in Fig. 2, where 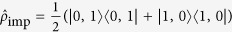. We emulate the operation of the quantum coprocessor by classically evaluating the quantum networks, and the classical exponential scaling limits our simulations to small systems. The self-consistency condition for the Bethe lattice calculated in the classical feedback loop is Λ_*σ*_(*t, t*′) = *v*(*t*)*G*_*σ*_(*t, t*′)*v*(*t*′), from which we obtain the SIAM coupling to bath *p* efficiently via a Cholesky decomposition 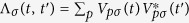, where * denotes complex conjugation (see Supplementary Material for details). The impurity site double occupancy 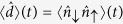 obtained from the self-consistent hybrid simulation is compared to the exact result in Fig. 3a and shows that Trotter errors do not noticeably affect our results.

Next we assume imperfect gates characterized by phase errors that are described by normally distributed random variables with zero mean^34^. We choose their standard deviations consistent with current experimental capabilities^1^,^13^,^35^ setting the single qubit error to *σ* = 10^−6^ and allowing MS gate errors *σ*_MS_ to vary between 0.1% and 10%. We obtain accurate results for the dynamics of the double occupancy even in the presence of gate errors. As shown in Fig. 3a the double occupation differs from the exact result by only ≈3% for *σ*_MS_ = 1%. For a smaller gate error of *σ*_MS_ = 0.1% the difference is insignificant up to *t* = 1.5/*v*_0_. In Fig. 3b we plot the error in the imaginary part of the lesser Green’s function 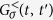 induced by imperfect gates. The diagonal values 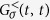, which determine time-local single-particle observables, are almost unaffected even for large MS gate errors. Gate errors in general make the Green’s function decay faster with *t* − *t*′ than in the ideal case and will thus affect unequal time correlation functions.

We further investigate the effect of imperfect gates by considering the impurity site coupled to two bath sites via constant *V*_*pσ*_(*t*). We find that the imaginary part of the mean field differs from the exact solution by a factor of approximately exp(−*η*|*t*′ − *t*|) as shown in Fig. 4a. The decay rate *η* increases with *σ*_MS_ as displayed in the inset of Fig. 4a. This numerical evidence suggests that gate errors have the same effect as smearing out the bath energies *ε*_*pσ*_(*t*) to a similar width *η*. The impurity model including errors would then be equivalent to the bath sites possessing a finite coherence time 1/*η*. Since the number of gates is ∝*N* we expect *η* to only depend weakly on *N*.

A bath site with coherence time 1/*η* can be modelled by allowing an ideal bath to incoherently exchange particles with a reservoir at an ‘error’ rate Γ = *η*. This exchange of particles modifies the bath’s Green’s function from its ideal value of *g*_*pσ*_(*t*,*t*′) = 1 and correspondingly modifies the relation between impurity bath couplings and mean field to ref. 24 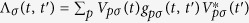. This relation does not necessarily allow for an exact solution for *V*_*pσ*_(*t*) even for large *N*. The effect of noise therefore limits the mean fields Λ_*σ*_(*t, t*′) that the bath sites can model.

We investigate if the noise induced by gate errors can be partly compensated by implementing self-consistency via this modified relation. For the non-interacting impurity with bath sites coupled to a particle reservoir we solve numerically for the bath Green’s functions *g*_*pσ*_(*t, t*′), exploiting the super-fermion formalism^36^ (see Supplementary Material). We minimize 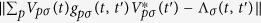 using the Frobenius norm over the *V*_*pσ*_(*t*) to obtain the hybridizations in the noisy system. This modification of the *classical* feedback loop significantly reduces the effect of gate errors as demonstrated in Fig. 4b, showing the reduction in average absolute error in the mean field Λ_*σ*_(*t, t*′). In the hybrid simulation scheme a slight modification of the quantum network shown in Fig. 2 allows the probe qubit to measure the bath Green’s functions, thus providing the information required for this noise-reduction scheme to be implemented.

Finally, we emphasize that our scheme works directly in the thermodynamic limit and, since it does not require a small expansion term, gives accurate results for all values of *U*, in particular for the challenging situation of intermediate interactions like the example *U* = 2*v*_0_ considered here. The number of available qubits only limits the number of bath sites that can be included in the simulation and hence the maximally reachable simulation time *t*_max_. Purely classical simulations are currently limited to approximately 25 bath sites^25^ and, because of fast growing SIAM entanglement^24^,^25^, scale exponentially with *t*_max_ despite efficiently implementing the feedback loop. Therefore, a quantum coprocessor with only about 50 qubits^1^ coupled to a classical feedback loop would be able to improve upon current purely classical algorithms. Our hybrid simulation scheme thus provides an interesting scientific application of next generation, possibly imperfect, quantum devices. While preparing this manuscript, we became aware of related work by B. Bauer *et al*.^37^.

## Methods

### Implementing the single-impurity Anderson model with the digital quantum simulator

To implement the SIAM in Eq. (2) in the main text with the digital quantum simulator, we first map the creation and annihilation operators in 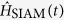 onto spin operators that act on the qubits in the coprocessor. This is achieved via the Jordan-Wigner transformation 
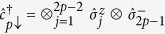
, 
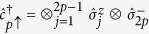
, and 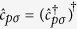 (we take *p* = 1 to be the impurity). Here, 
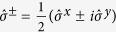
, and 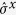, 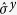, and 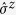 are the Pauli spin operators. The transformation maps *N* fermionic sites onto a string of 2*N* qubits such that two adjacent qubits represent one lattice site. The correspondences between the qubit states and fermionic states are |0, 0〉 = |vac〉, |1, 0〉 = |↓〉, |0, 1〉 = |↑〉, and |1, 1〉 = |↓↑〉.

To obtain the necessary quantum gates to approximate the unitary evolution operator we use a Trotter decomposition on the propagator 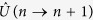 between each time *t*_*n*_ and *t*_*n*+1_ as 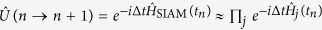, where 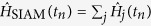. Each term 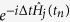 can be readily implemented using spin rotations 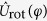 where *φ* is the angle of rotation, and multi-qubit Mølmer-Sørensen (MS) gates^30^,^31^, characterized by two phases *θ* and *ϕ* as 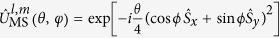, with 
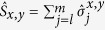
 (see Supplementary Material). Here, the MS gate acts on qubits *l, l* + 1, ⋯, *m*, and the phase *θ* controls the amount of entanglement, while varying *ϕ* allows a shift between a 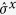 or a 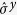 type gate.

### Measuring the impurity Green’s function with single-qubit interferometry

Using the Jordan-Wigner transformation, the lesser and greater impurity Green’s functions for each spin *σ* can be written as a sum of four expectation values of products of Pauli operators and evolution operators (see Supplementary Material). We use a single-qubit interferometry scheme^28^ to measure each of the expectation values *F*(*t, t*′) that constitute the Green’s function. We introduce a probe qubit which is coupled to the string of 2*N* system qubits. We assume that the probe qubit is prepared in the pure state |0〉, yielding the total system-probe density operator 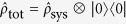. The combined system is then run through a Ramsey interferometer sequence, in which first a *π*/2 pulse (or Hadamard gate 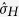) is applied to the probe qubit, the state of which will transform into the superposition 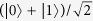. The two states in the superposition provide the necessary interference paths. Following the *π*/2 pulse, we apply the unitary evolution on the system of interest up to a certain time *t*′. The Pauli operators are then applied on the system as controlled quantum gates with either |0〉 or |1〉 as the control state. This is followed by evolution up to the final time *t*′, another controlled application of Pauli gates, and finally another *π*/2 pulse is applied on the probe qubit, bringing the interference paths together. The output state of the probe qubit at the end of the Ramsey sequence is given by


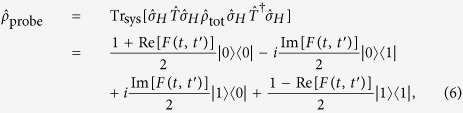


where 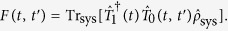 Here, the unitary operators 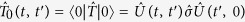 and 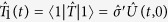, in which 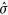 and 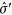 are Pauli operators or tensor products of Pauli operators (see Supplementary Material), act only on the system and not on the probe qubit. Note that we can write 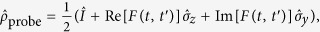 so that we have 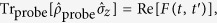 and 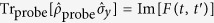. Therefore repeated measurements (which can be done in parallel) of the 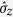 and 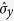 components of the probe qubit for all times *t*′ and *t* yields a contribution to the impurity Green’s function *G*_*σ*_(*t, t*′). For a spin-symmetric system, on the order of 80,000 measurements per time step are required. See Supplementary Material for details.

## Additional Information

**How to cite this article**: Kreula, J. M. *et al*. Non-linear quantum-classical scheme to simulate non-equilibrium strongly correlated fermionic many-body dynamics. *Sci. Rep.* **6**, 32940; doi: 10.1038/srep32940 (2016).

## Supplementary Material

Supplementary Information

## Figures and Tables

**Figure 1:**
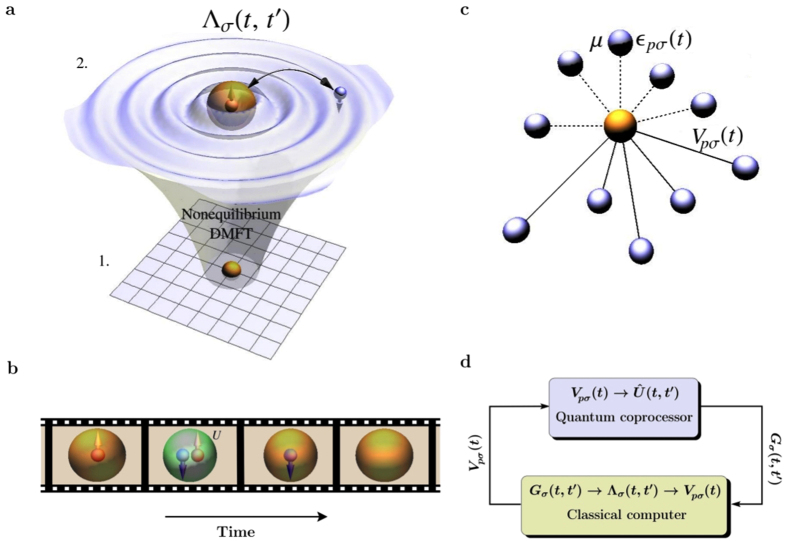
(**a)** In non-equilibrium DMFT a fermionic quantum lattice model is replaced by a single impurity site exchanging particles via a self-consistently determined time and spin dependent mean field Λ_*σ*_(*t, t*′). (**b)** This exchange of particles yields dynamical fluctuations of the impurity site occupation as a function of time shown here as |↑〉 → |↓↑〉 → |↓〉 →|vac〉. The onsite interaction *U* energetically penalises the doubly occupied state |↓↑〉. (**c)** The impurity-mean field interaction is mapped onto a SIAM with unitary evolution 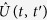. The energies of the non-interacting bath sites *p* are chosen 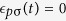 for *t* > 0 and their chemical potential is set *μ* = 0 in this work^24^. The impurity site exchanges fermions with time-dependent hybridization energies *V*_*pσ*_(*t*). (**d**) Quantum-classical hybrid simulation scheme: the SIAM dynamics for a given set of parameters *V*_*pσ*_(*t*) is implemented on a quantum coprocessor and yields the impurity Green’s function *G*_*σ*_(*t, t*′). The classical non-linear feedback loop takes *G*_*σ*_(*t, t*′) and calculates the mean field Λ_*σ*_(*t, t*′) from which a new set of *V*_*pσ*_(*t*) can be extracted. These parameters are then fed back into the quantum coprocessor and the loop is repeated until self-consistency is achieved.

**Figure 2:**
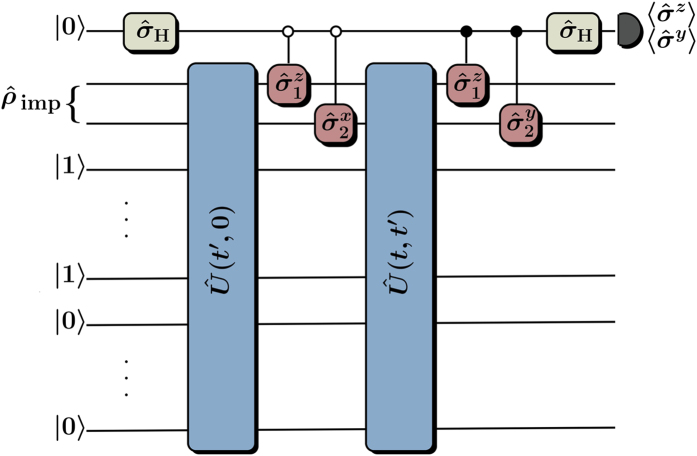
Coprocessor quantum network for measuring a contribution to *G*_*σ*_(*t, t′*) in the SIAM dynamics. This example network is given for the paramagnetic phase starting from the atomic limit, as considered in the main text and in ref. 24. A probe qubit (top line) is prepared in a symmetric superposition 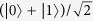 of computational basis states |0〉 and |1〉 by a Hadamard gate 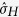. Here, 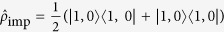, and the initial states of the bath sites (lines below the impurity) are set to either |0〉 or |1〉 using Jordan-Wigner transformed operators, following the standard scheme in ref. 24. After evolving the SIAM to time *t*′ according to 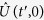 the probe qubit interacts with the impurity via controlled Pauli gates. A second set of controlled Pauli gates is applied after evolving the impurity to time *t*. The precise choice of Pauli gates selects different contributions to the Green’s function. After another Hadamard gate this contribution is encoded in the expectation values 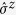 and 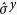 of the probe qubit, as discussed in Methods.

**Figure 3:**
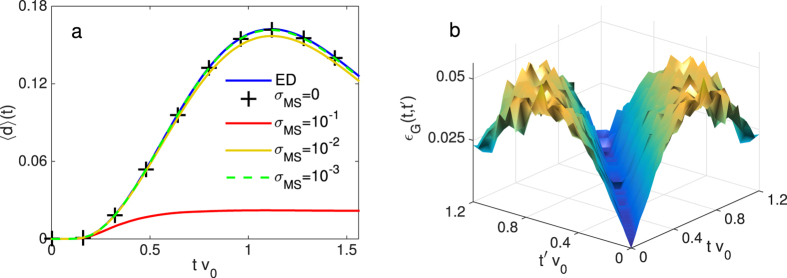
Hybrid non-equilibrium DMFT simulation results when dynamically increasing the Hubbard tunneling matrix element *v*(*t*) from 0 to *v*_0_ as described in the main text. We choose *U* = 2*v*_0_, Trotter steps Δ*t* = 0.04/*v*_0_ and couple the impurity site to *N* = 2 bath sites. (**a)** Impurity double occupation 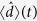 as a function of time *t*: numerically exact solution (blue solid curve), solution with Trotter errors (+), solutions including gate errors of *σ*_MS_ = 0.1% (green dashed curve), *σ*_MS_ = 1% (yellow solid curve), and *σ*_MS_ = 10% (red solid curve). (**b)** Absolute value of the difference 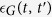 between the imaginary parts of the lesser Green’s function without gate errors and with gate errors of *σ*_MS_ = 1%. Results of calculations with gate errors are obtained by averaging over 128 realizations of the setup.

**Figure 4:**
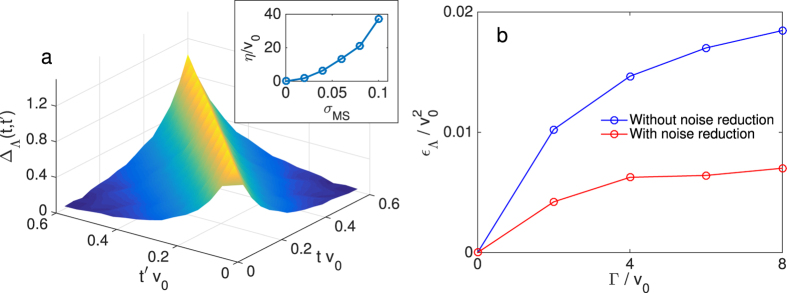
(**a**) Deviation of the mean field 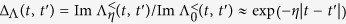, where 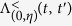 is the lesser component of the mean field, in the absence (presence) of gate errors (of *σ*_MS_ = 6%) for constant hybridizations and *U* = 2*v*_0_, *N* = 2 and averaged over 128 realizations. The inset shows the exponential decay rate *η* against two qubit error *σ*_MS_. (**b**) Average error in the self-consistent mean field 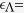

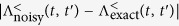
 for the non-interacting system with *N* = 10 noisy bath sites.
