# Supplementary material for: Non-linear quantum-classical scheme to simulate non-equilibrium strongly correlated fermionic many-body dynamics
Source: Sci Rep. 2016 Sep 9;6:32940. doi: 10.1038/srep32940 (PMC5016988; doi:10.1038/srep32940)
Supplement: Supplementary Information [file srep32940-s1.pdf]

Supplementary material to: "Non-linear quantum-classical scheme to simulate non-equilibrium strongly correlated fermionic many-body dynamics"

J. M. Kreula,[1] S. R. Clark,[2,3] and D. Jaksch[1,4]

[1] *Clarendon Laboratory, University of Oxford, Parks Road, Oxford OX1 3PU, United Kingdom*
[2] *Department of Physics, University of Bath, Claverton Down, Bath BA2 7AY, United Kingdom*
[3] *Max Planck Institute for the Structure and Dynamics of Matter, Hamburg, Germany*
[4] *Centre for Quantum Technologies, National University of Singapore, 3 Science Drive 2, Singapore 117543*

We provide background details on non-equilibrium dynamical mean-field theory and the setup that we study in the main text, and present the formulae needed for implementing the single-impurity Anderson model with qubits and on measuring the single-particle non-equilibrium Green function. We further elucidate the non-interacting system where we studied reducing the effects of a noisy bath.

I. NON-EQUILIBRIUM DYNAMICAL MEAN-FIELD THEORY

One of the simplest models to capture essential phenomena in strongly-correlated electron materials is the single-band Hubbard Hamiltonian

$$\hat{H}_{\text{Hubbard}} = -\sum_{\langle i,j\rangle\sigma} v_{ij}(t)\left(\hat{c}^{\dagger}_{i,\sigma}\hat{c}_{j,\sigma} + \text{h.c.}\right) + U(t)\sum_i \left(\hat{n}_{i,\uparrow} - \frac{1}{2}\right)\left(\hat{n}_{i,\downarrow} - \frac{1}{2}\right), \quad (1)$$

where $v_{ij}(t)$ is the tunnelling ('hopping') matrix element between nearest-neighbour sites i and j, and $U(t)$ is the on-site Coulomb repulsion. Here, we have assumed general time-dependent parameters due to the driving of material via, e.g., intense laser pulses[1]. Furthermore, $\hat{c}^{\dagger}_{i,\sigma}$ ($\hat{c}_{i,\sigma}$) is the creation (annihilation) operator for an electron with spin projection $\sigma = \uparrow, \downarrow$ at site i, while $\hat{n}_{i,\sigma} = \hat{c}^{\dagger}_{i,\sigma}\hat{c}_{i,\sigma}$ is the corresponding number operator.

Despite its apparent simplicity, the Hubbard model (1) is notoriously difficult to solve, even numerically, and especially in two dimensions where it may be relevant to high-T_c superconductivity. Fortunately, dynamical mean-field theory (DMFT)[2] and its extension to non-equilibrium problems[3] provide a means to compute local observables by circumventing the necessity of dealing directly with the Hubbard Hamiltonian. This is achieved by mapping it onto an impurity model, the solution of which is usually easier to obtain, albeit still a highly non-trivial computational task. The mapping is justified in the limit of infinite spatial dimensions, $d \to \infty$, (or infinite coordination, $z \to \infty$) by the collapse of the irreducible self-energy of the Hubbard model to only contributions emerging from strictly local skeleton diagrams which are identical to those of an impurity model. The collapse of the skeleton diagrams follows from the necessity to scale the hopping parameters as $v_{ij}(t) = v^*/\sqrt{z}$ to avoid a diverging average kinetic energy per lattice site and from simple power counting arguments. While describing the full Hubbard Hamiltonian with a single-impurity model is only an approximation in finite dimensions, it often relatively accurate already in three dimensions for certain lattice types.

The solution of the impurity model means essentially computing the local Green function

$$G_\sigma(t,t') = -i\langle \hat{c}_\sigma(t)\hat{c}^{\dagger}_\sigma(t')\rangle_{\hat{\mathcal{S}}_{\text{loc}}} = -i\frac{\text{Tr}\left\{\mathcal{T}_{\mathcal{C}}\left[\exp(\hat{\mathcal{S}}_{\text{loc}})\hat{c}_\sigma(t)\hat{c}^{\dagger}_\sigma(t')\right]\right\}}{\text{Tr}\left\{\mathcal{T}_{\mathcal{C}}\left[\exp(\hat{\mathcal{S}}_{\text{loc}})\right]\right\}}, \quad (2)$$

where $\mathcal{T}_{\mathcal{C}}$ is the contour-ordering operator on an 'L-shaped' Keldysh time-contour $\mathcal{C}$ (see Fig. 1). The local action $\hat{\mathcal{S}}_{\text{loc}}$ is given by[4]

$$\hat{\mathcal{S}}_{\text{loc}} = -i\int_{\mathcal{C}} dt\left[U(t)\left(\hat{n}_\uparrow(t) - \frac{1}{2}\right)\left(\hat{n}_\downarrow(t) - \frac{1}{2}\right) - \mu\sum_\sigma \hat{n}_\sigma(t)\right] - i\int_{\mathcal{C}} dt\int_{\mathcal{C}} dt' \sum_\sigma \Lambda_\sigma(t,t')\hat{c}^{\dagger}_\sigma(t)\hat{c}_\sigma(t'). \quad (3)$$

Here, μ is the chemical potential and Λ_σ is the *a priori* unknown hybridization function, or Weiss function, that describes the exchange of electrons between the impurity site with a bath of non-interacting electrons. The essential step in DMFT is the self-consistent determination of Λ_σ. For a Bethe lattice, which corresponds to a semi-elliptical density of states $D(\epsilon) = \sqrt{4v^2 - \epsilon^2}/(2\pi v^2)$, the DMFT self-consistency condition obtains a simple closed form. For time-dependent hoppings v, this reads

$$\Lambda_\sigma(t,t') = v(t)G_\sigma(t,t')v(t'). \quad (4)$$

The impurity action (3) can also be represented in a Hamiltonian form which permits the application of Hamiltonian-based numerical methods[4,5] to compute the local Green function. It also makes it possible to use the trapped-ion scheme for quantum simulations[6]. The impurity model that we address here is the single-impurity Anderson model (SIAM) given by

$$\hat{H}_{\text{SIAM}} = \hat{H}_{\text{loc}} + \hat{H}_{\text{bath}} + \hat{H}_{\text{hyb}}, \quad (5)$$

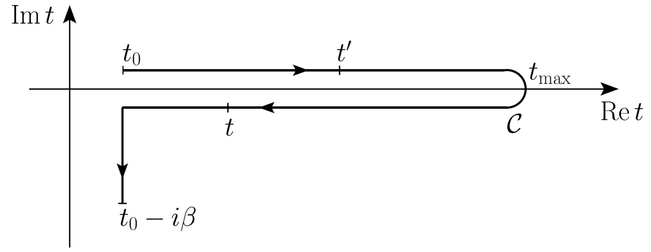

FIG. 1: Keldysh time-contour $\mathcal{C}$. It consists of two real-time branches between an initial time t_0 to final time $t_{\max}$, and an imaginary-time branch from t_0 to $t_0 - i\beta$, where β is the inverse temperature.

$$\hat{H}_{\text{loc}} = -\mu \sum_{\sigma} \hat{n}_{\sigma} + U(t) \left(\hat{n}_{\uparrow} - \frac{1}{2}\right) \left(\hat{n}_{\downarrow} - \frac{1}{2}\right), \quad (6)$$

$$\hat{H}_{\text{hyb}} = \sum_{p} \left(V_{p\sigma}(t) \hat{a}^{\dagger}_{\sigma} \hat{a}_{p\sigma} + \text{H.c.}\right), \quad (7)$$

$$\hat{H}_{\text{bath}} = \sum_{p,\sigma} \left[\epsilon_{p\sigma}(t) - \mu\right] \hat{a}^{\dagger}_{p\sigma} \hat{a}_{p\sigma}, \quad (8)$$

Here, $\hat{a}^{\dagger}_{\sigma}$ ($\hat{a}_{\sigma}$) is the creation (annihilation) operator for the impurity orbital, and $\hat{a}^{\dagger}_{p\sigma}$ ($\hat{a}_{p\sigma}$) for a bath orbital p. Further, $V_{p\sigma}(t)$ describes the hopping of electrons between the impurity and the bath, and $\epsilon_{p\sigma}(t)$ denotes the energy of the bath orbital p.

The SIAM Hamiltonian (5) corresponds to the correct DMFT action (3) if the parameters $V_{p\sigma}$ and $\epsilon_{p\sigma}(t)$ are chosen such that the relation

$$\Lambda^{\text{SIAM}}_{\sigma}(t,t') = \Lambda_{\sigma}(t,t') \quad (9)$$

is valid on the whole Keldysh contour $\mathcal{C}$. Here, the SIAM hybridization function has the expression[4]

$$\Lambda^{\text{SIAM}}_{\sigma}(t,t') = \sum_{p} V_{p\sigma}(t) g_{p\sigma}(t,t') V_{p\sigma}(t')^{*}, \quad (10)$$

where

$$g_{p\sigma}(t,t') = i \left[f(\epsilon_{p\sigma}(0) - \mu) - \Theta_{\mathcal{C}}(t,t')\right] e^{-i \int_{\mathcal{C}} d\bar{t}\, (\epsilon_{p\sigma}(\bar{t}) - \mu)} \quad (11)$$

is the non-interacting Green function for an isolated bath site, with $f(\epsilon) = 1/\left(\exp(\beta\epsilon) + 1\right)$ denoting the Fermi distribution function and $\Theta_{\mathcal{C}}(t,t')$ being the contour Heaviside function defined as

$$\Theta_{\mathcal{C}}(t,t') = \begin{cases} 1 & \text{if } t \geq_{\mathcal{C}} t' \\ 0 & \text{else.} \end{cases} \quad (12)$$

An essential part of the Hamiltonian-based DMFT scheme is the determination of the parameters $V_{p\sigma}(t)$ and $\epsilon_{p\sigma}(t)$ for a given hybridization function $\Lambda_{\sigma}(t,t')$. In what follows, we will relax the spin index σ for the hybridization function since below we will be dealing with a spin-symmetric set-up where both contributions are identical.

For non-equilibrium problems, it is useful introduce two distinct baths, with each having their own corresponding hybridization function[4]. The first bath, with hybridization Λ_{-}, includes those sites that are coupled to the impurity at $t = 0$. Often this first bath vanishes as $t \to \infty$. The second bath, Λ_{+}, builds up as time evolves, i.e., couples additional bath sites to the impurity for times $t > 0$. We will consider a system with no initial correlations ($\Lambda_{-} = 0$) in the next section, and focus only on the second bath, with Weiss function

$$\Lambda_{+}(t,t') = \sum_{p} V^{+}_{p}(t) g_{p}(t,t') V^{+}_{p}(t')^{*}. \quad (13)$$

Since all imaginary-time components, which account for initial correlations, vanish for Λ_{+}, we set $V^{+}_{p}(t = 0) = 0$ for all bath sites that are included in Λ_{+}. The time-dependence of the bath energies $\epsilon_{p}(t)$ can be absorbed in the time dependence of the hoppings $V^{+}_{p}(t)$, meaning that we are free to choose the evolution[4]

$$\epsilon_{p}(t) = \begin{cases} \epsilon_{p}(0) & \text{for } t = 0 \\ \epsilon(\infty) & \text{for } t > 0 \end{cases}, \quad (14)$$

where $\epsilon(\infty)$ is a constant. Moreover, since $\epsilon_{p}(0)$ is incorporated only in the Fermi functions $f[\pm(\epsilon_{p}(0) - \mu)]$ for $\Lambda^{</>}_{+}$, we can simply choose $\epsilon_{p}(0)$ such that $f(\epsilon_{p}(0) - \mu)$ is equal to 0 or 1. This is done in order to find a representation of the Weiss functions as

$$-i\Lambda^{<}_{+}(t,t') = \sum_{p \in B_{\text{occ}}} V^{+}_{p}(t) V^{+}_{p}(t')^{*}, \quad (15)$$

$$i\Lambda^{>}_{+}(t,t') = \sum_{p \in B_{\text{empty}}} V^{+}_{p}(t) V^{+}_{p}(t')^{*}, \quad (16)$$

in which B_{occ} and B_{empty} denote the sets of initially occupied and empty bath sites, respectively. Note that for a particle-hole symmetric system, $\Lambda^{<}_{+}(t,t') = \Lambda^{>}_{+}(t,t')^{*}$, which is satisfied if the occupied and empty bath sites come in pairs with complex conjugate hybridizations. Moreover, for a discretized time $t_n = n \times \Delta t \in [0, N \times \Delta t = t_{\max}]$, we have, e.g.,

$$(-i\Lambda^{<}_{+})_{nn'} = -i\Lambda^{<}_{+}(t_n, t_{n'}) = \sum_{p \in B_{\text{occ}}} V^{+}_{p}(t_n) V^{+}_{p}(t_{n'})^{*}, \quad (17)$$

which has the form of a Cholesky decomposition $(-i\Lambda^{<}_{+}) = VV^{\dagger}$ where V is a lower triangular matrix, the pth column of which gives the time-dependent hybridization to the bath orbital p. The use of Cholesky decomposition to the determine the hybridizations from the Weiss function allows us to adopt a time-propagation

scheme in which we do not update the whole Green and Weiss function matrices as time evolves but only the current time slice. In practice, since we only have a limited number of bath sites L, we employ an approximate representation of the Weiss function in which we obtain the evolution of the first L time-steps from the Cholesky decomposition, and for time steps greater than L we update a new column and row in the Weiss function matrix in a manner which minimizes the error in the approximate representation[4]. In the next section we present a test system and use the results of this section to determine the Weiss field self-consistently for the Hubbard model in an infinite-dimensional Bethe lattice.

II. THE SET-UP AND DMFT STEPS

We consider the time evolution of the infinite-dimensional Hubbard model with constant on-site interaction U and time-dependent hopping $v(t)$[4]. The hopping is turned on from the initial value $v = 0$ (i.e., the atomic limit) to the final value $v = v_0 = 1$, which we use as the unit of energy, with the profile

$$v(t) = \begin{cases} \frac{1}{2}\left[1 - \cos(\omega_0 t)\right] & \text{for } t < t_q \\ 1 & \text{for } t \geq t_q \end{cases}, \qquad (18)$$

where $\omega_0 = \pi/t_q$ and $t_q > 0$ is a suitable quench time. In our simulations we use $t_q = 0.25/v_0$. We assume a zero temperature initial state in the paramagnetic phase in the half-filled Bethe lattice. We then map the Hubbard model onto a SIAM. Since $v(t = 0) = 0$, Λ_- vanishes and the hybridization function is given by $\Lambda = \Lambda_+$. Since we have a spin- and a particle-hole symmetric system, the bath is represented with pairs of initially occupied and empty sites. We take the total number of bath sites to be $L_{\text{bath}} = 2L$, where L is the rank of the approximate representations of $-i\Lambda^<$ and $i\Lambda^>$. The initial ground state of the SIAM has an equal number of empty and doubly occupied bath sites with energies $\epsilon_{p\sigma} = 0$, and singly-occupied impurity which is spin-mixed with density matrix $\rho_0 = \left(|\uparrow\rangle\langle\uparrow| + |\downarrow\rangle\langle\downarrow|\right)/2$. To account for occupation of the impurity site, we consider two subsystems α and β, in which the impurity of the system α (β) is initially occupied by a single $\uparrow$-electron ($\downarrow$-electron). We then compute two impurity Green functions $G^{\alpha}_{\text{imp},\sigma}$ and $G^{\beta}_{\text{imp},\sigma}$ the average of which yields the local lattice Green function

$$G_{\text{loc},\sigma}(t, t') = \frac{1}{2}\left[G^{\alpha}_{\text{imp},\sigma} + G^{\beta}_{\text{imp},\sigma}\right], \qquad (19)$$

after self-consistency has been reached. Since we are considering the Hubbard model in the Bethe lattice, the DMFT self-consistency condition is given by Eq. (4).

The non-equilibrium DMFT steps to compute the single-particle lattice Green function for a maximum simulation time $t_{\max} = N \times \Delta t$ are then the following:

0. Choose an initial Green function g_0. For iteration $n = 1$, initialize the hybridization function as $\Lambda_1(t, t') = v(t) g_0(t, t') v(t')$, for $t, t' \leq t_{\max}$, where v is the hopping in the Hubbard Hamiltonian.

1. Use the Cholesky decomposition for Λ_n to obtain the hopping parameters $V_p(t)$ for the nth iteration.

2. Use exact diagonalization techniques to compute the impurity Green functions $G^{s}_{\text{imp},\sigma} = \Theta_{\mathcal{C}}(t, t') G^{s,>}_{\text{imp},\sigma}(t, t') + \Theta_{\mathcal{C}}(t', t) G^{s,<}_{\text{imp},\sigma}(t, t')$ for $s = \alpha$ and $s = \beta$, where

$$\begin{aligned} G^{s,>}_{\text{imp},\sigma}(t, t') &= -i\langle\psi^s_0|\hat{U}(0, t)\hat{c}_{1\sigma}\hat{U}(t, t')\hat{c}^{\dagger}_{1\sigma}\hat{U}(t', 0)|\psi^s_0\rangle, \\ G^{s,<}_{\text{imp},\sigma}(t, t') &= i\langle\psi^s_0|\hat{U}(0, t')\hat{c}^{\dagger}_{1\sigma}\hat{U}(t', t)\hat{c}_{1\sigma}\hat{U}(t, 0)|\psi^s_0\rangle, \\ \hat{U}(t, t') &= \mathcal{T} e^{-i\int_{t'}^{t} d\tau\, \hat{H}_{\text{SIAM}}(\tau)}. \end{aligned} \qquad (20)$$

Here, $|\psi^s_0\rangle$ is the initial (pure) state for system s, and $\mathcal{T}$ is the (usual) time-ordering operator. Use Eq. (19) to obtain the local lattice Green function G_n.

3. Use the DMFT self-consistency condition for the Bethe lattice $\Lambda_{n+1}(t, t') = v(t) G_n(t, t') v(t')$ to obtain the hybridization function for the next iteration.

4. Go to step 1 and iterate the steps until convergence is reached. The convergence variable can be, e.g., $\max|V_p(t) - V_{p,\text{prev}}(t)|$.

From the lattice Green function we can obtain single-particle observables. In addition to the Green function, in the time-evolution we can calculate the time-dependent double occupation $\langle d(t)\rangle = \langle \hat{n}_{1\uparrow}\hat{n}_{1\downarrow}\rangle$ which is also averaged over the systems α and β.

III. JORDAN–WIGNER TRANSFORMATION APPLIED TO THE SINGLE-IMPURITY ANDERSON MODEL

The aim of the main article is to show how such DMFT steps as described above could be performed on a trapped-ion quantum computer in conjunction with a classical feedback loop. To this end, we must represent the SIAM Hamiltonian (5) with $\mu = 0$ and $\epsilon_{p\sigma} = 0$ in terms of spin operators that operate on the qubits. This is achieved via the Jordan–Wigner transformation, in which we map a string of N fermions onto a string of $2N$ qubits. The relation between the fermionic creation and annihilation operators and the spin operators reads

$$\hat{a}^{\dagger}_{p\downarrow} = \hat{\sigma}^z_1 \otimes \cdots \otimes \hat{\sigma}^z_{2p-2} \otimes \hat{\sigma}^-_{2p-1}, \qquad (21)$$

$$\hat{a}^{\dagger}_{p\uparrow} = \hat{\sigma}^z_1 \otimes \cdots \otimes \hat{\sigma}^z_{2p-1} \otimes \hat{\sigma}^-_{2p}, \qquad (22)$$

$$\hat{a}_{p\sigma} = (\hat{a}^{\dagger}_{p\sigma})^{\dagger}, \qquad (23)$$

where $\hat{\sigma}^{\pm} = \frac{1}{2}(\hat{\sigma}^x \pm i\hat{\sigma}^y)$, and $\hat{\sigma}^x$, $\hat{\sigma}^y$, and $\hat{\sigma}^z$ are the Pauli spin operators.

We apply the transformations (21)-(23) to the SIAM Hamiltonian. The interaction term becomes

$$U(t)\left(\hat{n}_\uparrow - \frac{1}{2}\right)\left(\hat{n}_\downarrow - \frac{1}{2}\right) = \frac{1}{4}U(t)\hat{\sigma}_1^z \otimes \hat{\sigma}_2^z, \qquad (24)$$

while the hybridization terms read

$$V_{p\downarrow}\hat{a}^\dagger_{1\downarrow}\hat{a}_{p\downarrow} + \text{H.c.} = \frac{1}{2}\text{Re}(V_{p\downarrow})\left(\hat{\sigma}_1^x \otimes \hat{\sigma}_2^z \otimes \cdots \otimes \hat{\sigma}_{2p-2}^z \otimes \hat{\sigma}_{2p-1}^x + \hat{\sigma}_1^y \otimes \hat{\sigma}_2^z \otimes \cdots \otimes \hat{\sigma}_{2p-2}^z \otimes \hat{\sigma}_{2p-1}^y\right)$$
$$+ \frac{1}{2}\text{Im}(V_{p\downarrow})\left(\hat{\sigma}_1^y \otimes \hat{\sigma}_2^z \otimes \cdots \otimes \hat{\sigma}_{2p-2}^z \otimes \hat{\sigma}_{2p-1}^x - \hat{\sigma}_1^x \otimes \hat{\sigma}_2^z \otimes \cdots \otimes \hat{\sigma}_{2p-2}^z \otimes \hat{\sigma}_{2p-1}^y\right), \qquad (25)$$

$$V_{p\uparrow}\hat{a}^\dagger_{1\uparrow}\hat{a}_{p\uparrow} + \text{H.c.} = \frac{1}{2}\text{Re}(V_{p\uparrow})\left(\hat{\sigma}_2^x \otimes \hat{\sigma}_3^z \otimes \cdots \otimes \hat{\sigma}_{2p-1}^z \otimes \hat{\sigma}_{2p}^x + \hat{\sigma}_2^y \otimes \hat{\sigma}_3^z \otimes \cdots \otimes \hat{\sigma}_{2p-1}^z \otimes \hat{\sigma}_{2p}^y\right)$$
$$+ \frac{1}{2}\text{Im}(V_{p\uparrow})\left(\hat{\sigma}_2^y \otimes \hat{\sigma}_3^z \otimes \cdots \otimes \hat{\sigma}_{2p-1}^z \otimes \hat{\sigma}_{2p}^x - \hat{\sigma}_2^x \otimes \hat{\sigma}_3^z \otimes \cdots \otimes \hat{\sigma}_{2p-1}^z \otimes \hat{\sigma}_{2p}^y\right). \qquad (26)$$

In order to implement the time-evolution operator in an experiment, we use the Trotter decomposition

$$e^{-i\delta t\sum_{j=1}^N \hat{h}_j} \approx \prod_{j=1}^N e^{-i\delta t\hat{h}_j}, \qquad (27)$$

in which each of the terms on the right hand side can be implemented with the help of Mølmer–Sørensen gates and local and global rotations, as described in the next section.

IV. IMPLEMENTING THE SIAM HAMILTONIAN WITH MØLMER–SØRENSEN GATES

Each exponent that consists of tensor products of k Pauli operators can be implemented (up to local rotations) with a Mølmer–Sørensen gate acting on the k qubits, one local gate acting on a single qubit, and the inverse Mølmer–Sørensen gate[7,8]. For example, we have the decomposition

$$\hat{U} = \hat{U}_{\text{MS}}^{1,k}\left(-\frac{\pi}{2},0\right)\hat{U}_{1,\text{loc}}(\phi)\hat{U}_{\text{MS}}^{1,k}\left(\frac{\pi}{2},0\right)$$
$$= \exp\left(i\phi\sigma_1^z \otimes \sigma_2^x \otimes \sigma_3^x \otimes \cdots \otimes \sigma_k^x\right), \qquad (28)$$

where the Mølmer–Sørensen gate is given by

$$\hat{U}_{\text{MS}}^{l,m}(\theta,\phi) = \exp\left[-i\frac{\theta}{4}\left(\cos\phi\,\hat{S}_x + \sin\phi\hat{S}_y\right)^2\right], \qquad (29)$$

with $\hat{S}_{x,y} = \sum_{j=l}^m \hat{\sigma}_j^{x,y}$. The local gate in Eq. (28) reads

$$\hat{U}_{j,\text{loc}}(\phi) = \begin{cases} \exp(-i\phi\sigma_j^z) & \text{for } k = 4n-1 \\ \exp(i\phi\sigma_j^z) & \text{for } k = 4n+1 \\ \exp(-i\phi\sigma_j^y) & \text{for } k = 4n-2 \\ \exp(i\phi\sigma_j^y) & \text{for } k = 4n \end{cases}, \quad n \in \mathbb{N}, \qquad (30)$$

To implement a string of $\hat{\sigma}^y$ gates instead of $\hat{\sigma}^x$, we use a different Mølmer–Sørensen gate, yielding the decomposition

$$\hat{U} = \hat{U}_{\text{MS}}^{1,k}\left(-\frac{\pi}{2},\frac{\pi}{2}\right)\hat{U}_{1,\text{loc}}(\phi)\hat{U}_{\text{MS}}^{1,k}\left(\frac{\pi}{2},\frac{\pi}{2}\right)$$
$$= \exp\left(i\phi\sigma_1^z \otimes \sigma_2^y \otimes \sigma_3^y \otimes \cdots \otimes \sigma_k^y\right), \qquad (31)$$

with the local gate

$$\hat{U}_{j,\text{loc}}(\phi) = \begin{cases} \exp(-i\phi\sigma_j^z) & \text{for } k = 4n-1 \\ \exp(i\phi\sigma_j^z) & \text{for } k = 4n+1 \\ \exp(i\phi\sigma_j^x) & \text{for } k = 4n-2 \\ \exp(-i\phi\sigma_j^x) & \text{for } k = 4n \end{cases}, \quad n \in \mathbb{N}. \qquad (32)$$

Any of the gates from Eqs. (25) and (26) can be obtained from Eqs. (28) and (31) by applying additional local rotations. For instance,

$$\exp\left(i\phi\hat{\sigma}_2^x\otimes\hat{\sigma}_3^z\otimes\cdots\otimes\hat{\sigma}_{2p-1}^z\otimes\hat{\sigma}_{2p}^x\right)=\exp\left(i\frac{\pi}{4}\sum_{j=4}^{2p-1}\hat{\sigma}_j^y\right)\hat{U}_{\rm MS}^{2,2p}\left(-\frac{\pi}{2},0\right)\hat{U}_{3,\rm loc}(\phi)\hat{U}_{\rm MS}^{2,2p}\left(\frac{\pi}{2},0\right)\exp\left(-i\frac{\pi}{4}\sum_{j=4}^{2p-1}\hat{\sigma}_j^y\right),\tag{33}$$

where $\hat{U}_{3,\rm loc}(\phi)=\exp\left(-i\phi\hat{\sigma}_3^z\right)$ for even p, and $\hat{U}_{3,\rm loc}(\phi)=\exp\left(i\phi\hat{\sigma}_3^z\right)$ for odd p, with $\phi=-\frac{1}{2}\delta t\mathrm{Re}(V_{p\uparrow})$. Similarly, e.g.,

$$\begin{aligned}&\exp\left(i\phi\hat{\sigma}_1^x\otimes\hat{\sigma}_2^z\otimes\cdots\otimes\hat{\sigma}_{2p-2}^z\otimes\hat{\sigma}_{2p-1}^y\right)\\&=\exp\left(i\frac{\pi}{4}\hat{\sigma}_1^z\right)\exp\left(-i\frac{\pi}{4}\sum_{j=3}^{2p-2}\hat{\sigma}_j^y\right)\hat{U}_{\rm MS}^{1,2p-1}\left(-\frac{\pi}{2},\frac{\pi}{2}\right)\hat{U}_{2,\rm loc}(\phi)\hat{U}_{\rm MS}^{1,2p-1}\left(\frac{\pi}{2},\frac{\pi}{2}\right)\exp\left(i\frac{\pi}{4}\sum_{j=3}^{2p-2}\hat{\sigma}_j^y\right)\exp\left(-i\frac{\pi}{4}\hat{\sigma}_1^z\right),\end{aligned}\tag{34}$$

where $\hat{U}_{2,\rm loc}(\phi)=\exp\left(-i\phi\hat{\sigma}_2^z\right)$ for even p, and $\hat{U}_{2,\rm loc}(\phi)=\exp\left(i\phi\hat{\sigma}_2^z\right)$ for odd p, with $\phi=\frac{1}{2}\delta t\mathrm{Im}(V_{p\downarrow})$.

V. MEASURING THE LOCAL GREEN FUNCTION

An essential part of the scheme is the determination of the local non-equilibrium Green function. In this section, we propose an experimental scheme to measure it with trapped ions. We again apply the Jordan–Wigner transformations on the $\hat{c}$-operators and obtain the following expressions for the different components of the Green function

$$\begin{aligned}G_{1\uparrow}^{s,>}(t,t')=&-\frac{i}{4}\Big(\langle\psi_0^s|\hat{U}(0,t)(\hat{\sigma}_1^z\otimes\hat{\sigma}_2^x)\hat{U}(t,t')(\hat{\sigma}_1^z\otimes\hat{\sigma}_2^x)\hat{U}(t',0)|\psi_0^s\rangle-i\langle\psi_0^s|\hat{U}(0,t)(\hat{\sigma}_1^z\otimes\hat{\sigma}_2^x)\hat{U}(t,t')(\hat{\sigma}_1^z\otimes\hat{\sigma}_2^y)\hat{U}(t',0)|\psi_0^s\rangle\\&+i\langle\psi_0^s|\hat{U}(0,t)(\hat{\sigma}_1^z\otimes\hat{\sigma}_2^y)\hat{U}(t,t')(\hat{\sigma}_1^z\otimes\hat{\sigma}_2^x)\hat{U}(t',0)|\psi_0^s\rangle+\langle\psi_0^s|\hat{U}(0,t)(\hat{\sigma}_1^z\otimes\hat{\sigma}_2^y)\hat{U}(t,t')(\hat{\sigma}_1^z\otimes\hat{\sigma}_2^y)\hat{U}(t',0)|\psi_0^s\rangle\Big),\end{aligned}\tag{35}$$

$$\begin{aligned}G_{1\downarrow}^{s,>}(t,t')=&-\frac{i}{4}\Big(\langle\psi_0^s|\hat{U}(0,t)\hat{\sigma}_1^x\hat{U}(t,t')\hat{\sigma}_1^x\hat{U}(t',0)|\psi_0^s\rangle-i\langle\psi_0^s|\hat{U}(0,t)\hat{\sigma}_1^x\hat{U}(t,t')\hat{\sigma}_1^y\hat{U}(t',0)|\psi_0^s\rangle\\&+i\langle\psi_0^s|\hat{U}(0,t)\hat{\sigma}_1^y\hat{U}(t,t')\hat{\sigma}_1^x\hat{U}(t',0)|\psi_0^s\rangle+\langle\psi_0^s|\hat{U}(0,t)\hat{\sigma}_1^y\hat{U}(t,t')\hat{\sigma}_1^y\hat{U}(t',0)|\psi_0^s\rangle\Big),\end{aligned}\tag{36}$$

$$\begin{aligned}G_{1\uparrow}^{s,<}(t,t')=&\frac{i}{4}\Big(\langle\psi_0^s|\hat{U}(0,t')(\hat{\sigma}_1^z\otimes\hat{\sigma}_2^x)\hat{U}(t',t)(\hat{\sigma}_1^z\otimes\hat{\sigma}_2^x)\hat{U}(t,0)|\psi_0^s\rangle+i\langle\psi_0^s|\hat{U}(0,t')(\hat{\sigma}_1^z\otimes\hat{\sigma}_2^x)\hat{U}(t',t)(\hat{\sigma}_1^z\otimes\hat{\sigma}_2^y)\hat{U}(t,0)|\psi_0^s\rangle\\&-i\langle\psi_0^s|\hat{U}(0,t')(\hat{\sigma}_1^z\otimes\hat{\sigma}_2^y)\hat{U}(t',t)(\hat{\sigma}_1^z\otimes\hat{\sigma}_2^x)\hat{U}(t,0)|\psi_0^s\rangle+\langle\psi_0^s|\hat{U}(0,t')(\hat{\sigma}_1^z\otimes\hat{\sigma}_2^y)\hat{U}(t',t)(\hat{\sigma}_1^z\otimes\hat{\sigma}_2^y)\hat{U}(t,0)|\psi_0^s\rangle\Big),\end{aligned}\tag{37}$$

$$\begin{aligned}G_{1\downarrow}^{s,<}(t,t')=&\frac{i}{4}\Big(\langle\psi_0^s|\hat{U}(0,t')\hat{\sigma}_1^x\hat{U}(t',t)\hat{\sigma}_1^x\hat{U}(t,0)|\psi_0^s\rangle+i\langle\psi_0^s|\hat{U}(0,t')\hat{\sigma}_1^x\hat{U}(t',t)\hat{\sigma}_1^y\hat{U}(t,0)|\psi_0^s\rangle\\&-i\langle\psi_0^s|\hat{U}(0,t')\hat{\sigma}_1^y\hat{U}(t',t)\hat{\sigma}_1^x\hat{U}(t,0)|\psi_0^s\rangle+\langle\psi_0^s|\hat{U}(0,t')\hat{\sigma}_1^y\hat{U}(t',t)\hat{\sigma}_1^y\hat{U}(t,0)|\psi_0^s\rangle\Big),\end{aligned}\tag{38}$$

In Eqs. (35)-(38), all time-evolution operators $\hat{U}(t,0)$, etc, correspond to a sequence of quantum gates obtained in the previous section.

To measure each of the summands in Eqs. (35)-(38), we introduce a probe qubit[9] which we couple to the system of interest. We assume that the probe qubit is prepared in the pure state $|0\rangle$, yielding the total density operator $\hat{\rho}_{\rm tot}=\hat{\rho}_{\rm sys}\otimes|0\rangle\langle 0|$. The combined system is then run

through a Ramsey interferometer sequence described by a quantum circuit in which we first apply a Hadamard gate $\hat{\sigma}_H$ ($\pi/2$ pulse) on the probe qubit, followed by unitary evolution of the system of interest, followed by a controlled application of Pauli gates, evolution up to the final time, another controlled application of Pauli gates, and ending with another Hadamard gate on the probe qubit (see Fig. 2 of the main text). The output state of the qubit at the end of the Ramsey sequence is given by

$$\begin{aligned}\hat{\rho}_{\rm probe} =& \mathrm{Tr}_{\rm sys}\left[\hat{\sigma}_H\hat{T}\hat{\sigma}_H\hat{\rho}_{\rm tot}\hat{\sigma}_H\hat{T}^\dagger\hat{\sigma}_H\right] \\ =& \frac{1+\mathrm{Re}[F(t,t')]}{2}|0\rangle\langle 0| - i\frac{\mathrm{Im}[F(t,t')]}{2}|0\rangle\langle 1| \\ &+ i\frac{\mathrm{Im}[F(t,t')]}{2}|1\rangle\langle 0| + \frac{1-\mathrm{Re}[F(t,t')]}{2}|1\rangle\langle 1|,\end{aligned} \tag{39}$$

where $F(t,t') = \mathrm{Tr}_{\rm sys}\left[\hat{T}_1^\dagger(t)\hat{T}_0(t,t')\hat{\rho}_{\rm sys}\right]$ corresponds to one of the summands in Eqs. (35)-(38). Here, the unitary operators $\hat{T}_0(t,t') = \langle 0|\hat{T}|0\rangle = \hat{U}(t,t')\hat{\sigma}\hat{U}(t',0)$ and $\hat{T}_1(t) = \langle 1|\hat{T}|1\rangle = \hat{\sigma}'\hat{U}(t,0)$, in which $\hat{\sigma}$ and $\hat{\sigma}'$ are Pauli operators or tensor products of Pauli operators according to Eqs. (35)-(38), act only on the system and not on the probe qubit. For example, the network in Fig. 2 of the main text corresponds to the case $\hat{\sigma} = \hat{\sigma}_1^z \otimes \hat{\sigma}_2^x$ and $\hat{\sigma}' = \hat{\sigma}_1^z \otimes \hat{\sigma}_2^y$. Note that

$$\hat{\rho}_{\rm probe} = \frac{1}{2}\left(\hat{I} + \mathrm{Re}[F(t,t')]\hat{\sigma}_z + \mathrm{Im}[F(t,t')]\hat{\sigma}_y\right), \tag{40}$$

where $\hat{I}$ is the identity operator, so that we have

$$\mathrm{Tr}_{\rm probe}\left[\hat{\rho}_{\rm probe}\hat{\sigma}_z\right] = \mathrm{Re}[F(t,t')], \tag{41}$$

and

$$\mathrm{Tr}_{\rm probe}\left[\hat{\rho}_{\rm probe}\hat{\sigma}_y\right] = \mathrm{Im}[F(t,t')], \tag{42}$$

which are then experimentally measurable quantities.

To give a rough estimate on the number of measurements required in an experiment, we consider the probe to be in the superposition $\frac{1}{\sqrt{2}}(|0\rangle + |1\rangle)$ which is the state with maximal uncertainty in the measurement outcome. Thus, the measurement of the σ^z component yields either -1 or +1 with probability $\frac{1}{2}$. This random variable then follows a two-point distribution with parameters $p = 0.5$, $q = 1 - p = 0.5$, and variance $\sigma^2 = 1$. The mean of the σ^z component in this state is zero. To obtain this mean with a standard error of the mean $\epsilon = \sigma/\sqrt{n}$ requires $n = \sigma^2/\epsilon^2$ projective measurements for each contribution to the Green function. For example for $\epsilon = 0.02$ we would need about $2\times2\times2\times4\times2\times2\,500 = 160\,000$ [2 systems (α and β), 2 spins, lesser and greater Green function, 4 terms per Green function, 2 expectation values to be measured, and 2 500 measurements for each expectation value] measurements per time step, and this number scales quadratically with the number of points in the time grid. However, if we consider a spin-symmetric system as above, where we have the symmetries $G_{1\sigma}^{\alpha(\beta),</>} = G_{1\bar{\sigma}}^{\beta(\alpha),</>}$, we only need to measure half of the Green functions above. Note that all measurements can be done in parallel.

VI. OUTLINE OF THE CLASSICAL SIMULATIONS OF THE HYBRID DEVICE

We perform classical simulations of the single-qubit interferometer described in the previous section. In the actual hybrid device, the single-qubit interferometry would be done experimentally, and here we try to mimic the experimental procedure.

We consider the first L time steps, where L is the half the number of bath sites. We first obtain some initial guess hybridization parameters $V_{p\sigma}^{(0)}(t)$, where $t = 0, \Delta t, \ldots, L\Delta$. Using $V_{p\sigma}^{(0)}(t)$ we construct *imperfect* quantum gates $\hat{U}_{\rm rot}(\varphi+\epsilon)$ and $\hat{U}_{\rm MS}^{l,m}(\theta+\epsilon_{\rm MS1}, \phi+\epsilon_{\rm MS2})$, where ϵ, $\epsilon_{\rm MS1}$, and $\epsilon_{\rm MS2}$ are normally distributed random variables with zero mean and standard deviations σ, $\sigma_{\rm MS1}$, and $\sigma_{\rm MS2}$, respectively. These quantum gates yield the Trotterized unitary evolution operator $\hat{U}(t,t')$, where $t, t' = 0, \Delta t, \ldots, L\Delta$. We use this evolution operator to compute the ($t = m\Delta t, t' = n\Delta t$)-point ($m, n \le L$) of $F(t,t')$ from $\mathrm{Tr}_{\rm probe}\left[\hat{\rho}_{\rm probe}\hat{\sigma}_z\right]$ and $\mathrm{Tr}_{\rm probe}\left[\hat{\rho}_{\rm probe}\hat{\sigma}_y\right]$ as explained in the previous section, and we average the results over several realizations to gather error statistics. After going through all the possible combinations of the controlled $\hat{\sigma}_1^x$ ($\hat{\sigma}_1^z\otimes\hat{\sigma}_2^x$) and $\hat{\sigma}_1^y$ ($\hat{\sigma}_1^z\otimes\hat{\sigma}_2^y$) gates according to Eqs. (35)-(38), we obtain $G_{\downarrow(\uparrow)}(t = m\Delta t, t' = n\Delta t)$.

However, we interpret the computation of the point ($t = m\Delta t, t' = n\Delta t$) as a measurement which collapses the state of the system, and we cannot store any information of the state at this time instant in memory, since we don't want to re-use any of the obtained wave functions later to avoid correlating the errors between different points in the Green function. We compute these points from independent realizations instead. This way we make our classical simulations to follow what one would do in an experiment. This means that in order to compute another point ($t = (m+1)\Delta t, t' = n\Delta t$) or ($t = m\Delta t, t' = (n+1)\Delta t$), we have to propagate again from the origin ($t = 0, t' = 0$) to the desired point and again average over several realizations. This procedure is repeated until we have obtained all the points of $G_\sigma(t,t')$ until ($t = L\Delta t, t' = L\Delta t$). This concludes the 'experimental', or quantum, part of the first L time steps in the *first* iteration of the DMFT self-consistency loop.

The obtained $G_\sigma(t,t')$ is then used in the classical computer to produce the hybridization function $\Lambda_\sigma(t,t') = v(t)G_\sigma(t,t')v(t')$. In the first L time steps, we have enough parameters to do a Cholesky decomposition of $\Lambda_\sigma(t,t')$ to obtain new hybridizations $V_{p\sigma}^{(1)}(t)$, which are used for updating $\hat{U}_{\rm rot}(\varphi+\epsilon)$ and $\hat{U}_{\rm MS}^{l,m}(\theta+\epsilon_{\rm MS1}, \phi+\epsilon_{\rm MS2})$. This begins the *second* iteration of the DMFT self-consistency loop where use the

updated quantum gates to again 'measure' $G_\sigma(t,t')$ using the steps described above, always starting from the origin to compute one point in the time grid and averaging over several realizations. This non-linear process of 'measuring' $G_\sigma(t,t')$ and using Cholesky decomposition of $\Lambda_\sigma(t,t')$ to update $V_{p\sigma}(t)$ is repeated until $|V_{p\sigma}^{(n)}(t) - V_{p\sigma}^{(n-1)}(t)| < \delta$ where δ is a predetermined error threshold.

For the time steps $L+1,\ldots,N$ with $t_{\max} = N\Delta t$, we adopt the 'time slicing' scheme of Ref.[4], where we iterate one time step $M > L$ to self-consistency before moving to $M+1$. In the classical part of the hybrid device, we utilize a simple minimizer step[4] to update only $V_{p\sigma}(M\Delta t)$ while keeping the previously obtained $V_{p\sigma}(K\Delta t)$ ($K < M$) fixed. However, again when we want to reach the Mth time step in the time grid, we have to start propagating from the origin.

Mimicking the experiment to this level makes our classical simulation very difficult. Thus, our simulations are limited to small system sizes and relatively short time scales.

VII. NON-INTERACTING SYSTEM AND ERROR CORRECTION

The non-interacting impurity system comprises of SIAM Hamiltonian (5) with $U = 0$. We take each bath site as being independently coupled to a thermal reservoir to which it can incoherently exchange electrons with. This is described within the quantum master equation approach where the density operator $\hat{\rho}(t)$ of the full system obeys

$$\begin{aligned}\frac{\mathrm{d}}{\mathrm{d}t}\hat{\rho}(t) =& - i[\hat{H}_{\mathrm{SIAM}}, \hat{\rho}(t)] \\ &+ \sum_{p>0,\sigma} \Gamma_p^- [2\hat{c}_{p\sigma}\hat{\rho}(t)\hat{c}_{p\sigma}^\dagger - \hat{\rho}(t)\hat{c}_{p\sigma}^\dagger\hat{c}_{p\sigma} - \hat{c}_{p\sigma}^\dagger\hat{c}_{p\sigma}\hat{\rho}(t)] \\ &+ \sum_{p>0,\sigma} \Gamma_p^+ [2\hat{c}_{p\sigma}^\dagger\hat{\rho}(t)\hat{c}_{p\sigma} - \hat{\rho}(t)\hat{c}_{p\sigma}\hat{c}_{p\sigma}^\dagger - \hat{c}_{p\sigma}\hat{c}_{p\sigma}^\dagger\hat{\rho}(t)],\end{aligned}$$

where $\Gamma_p^\pm$ are the rates of electron ejection ($-$) and injection ($+$) to bath site p. In the case of no impurity coupling $V_{p\sigma}(t) = 0$ the noise on each bath site will drive their occupancies to a steady-state value of $n_p(\infty) = \Gamma_p^+/(\Gamma_p^- + \Gamma_p^+)$.

Since this model is non-interacting and has Lindblad noise terms which are linear in the electron creation and annihilation operators the master equation can be solved exactly using the so-called super-fermion formalism[10]. Here we use this approach to compute the impurity single-particle Green functions

$$\begin{aligned}G_\sigma^>(t,t') &= i\mathrm{Tr}[\hat{\rho}_0\hat{c}_{1\sigma}^\dagger(t')\hat{c}_{1\sigma}(t)], \\ G_\sigma^<(t,t') &= -i\mathrm{Tr}[\hat{\rho}_0\hat{c}_{1\sigma}(t)\hat{c}_{1\sigma}^\dagger(t')],\end{aligned}$$

for this system given an initial density operator $\hat{\rho}_0$. We focused on a quench of the Hubbard hopping parameter $v(t)$ given by Eq. (18). The initial density operator $\hat{\rho}_0$ was again chosen to model a $T = 0$ half-filled paramagnetic phase[4], where $\mu = 0$, with the impurity being in a singly occupied spin-mixed state $\frac{1}{2}(|\uparrow\rangle\langle\uparrow| + |\downarrow\rangle\langle\downarrow|)$, along with half the bath sites were doubly occupied $|\uparrow\downarrow\rangle$, and the other half empty $|0\rangle$. The dissipation in the bath was taken to have $\Gamma_p^\pm = \Gamma$ so that the steady-state density of the system remains a constant unit-filling. We take the bath energies to $\epsilon_{p\sigma}(t) = 0$ throughout.

Using the calculated $G_\sigma^{\gtrless}(t,t')$ the non-equilibrium DMFT self-consistency loop was solved using (i) the standard Cholesky time-slicing proposed for a noiseless system[4], explained after Eq. (17), and (ii) using a fitting procedure which attempts to correct for the effects of the bath noise. We solve numerically for the bath Green functions $g_{p\sigma}(t,t')$ using the super-fermion approach[10]. To implement a noise-reduction scheme, we minimize $\left\|\sum_p V_{p\sigma}(t)g_{p\sigma}(t,t')V_{p\sigma}(t') - \Lambda_\sigma(t,t')\right\|_F^2$ ($\|\cdot\|_F$ is the Frobenius norm) over the $V_{p\sigma}(t)$ to obtain the hybridizations corresponding to a noisy system. It is often useful to include a multiplying function of the form $f(t,t') = \exp(-\mu|t-t'|)$ in the cost function to aid the convergence of the minimiser.

1 Wall, S. *et al.* Quantum interference between charge excitation paths in a solid-state Mott insulator. *Nature Phys.* **7**, 114–118 (2011).

2 Georges, A., Kotliar, G., Krauth, W. & Rozenberg, M. J. Dynamical mean-field theory of strongly correlated fermion systems and the limit of infinite dimensions. *Rev. Mod. Phys.* **68**, 13 (1996).

3 Aoki, H. *et al.* Nonequilibrium dynamical mean-field theory and its applications. *Rev. Mod. Phys.* **86**, 779–837 (2014).

4 Gramsch, C., Balzer, K., Eckstein, M. & Kollar, M. Hamiltonian-based impurity solver for nonequilibrium dynamical mean-field theory. *Phys. Rev. B* **88**, 235106 (2013).

5 Wolf, F. A., McCulloch, I. P. & Schollwöck, U. Solving nonequilibrium dynamical mean-field theory using matrix product states. *Phys. Rev. B* **90**, 235131 (2014).

6 Blatt, R. & Roos, C. F. Quantum simulations with trapped ions. *Nature Phys.* **8**, 277–284 (2012).

7 Casanova, J., Mezzacapo, A., Lamata, L. & Solano, E. Quantum simulation of interacting fermion lattice models in trapped ions. *Phys. Rev. Lett.* **108**, 190502 (2012).

8 Müller, M., Hammerer, K., Zhou, Y. L., Roos, C. F. & Zoller, P. Simulating open quantum systems: from many-

body interactions to stabilizer pumping. *New J. Phys.* **13**, 085007 (2011).

[9] Dorner, R. *et al.* Extracting quantum work statistics and fluctuation theorems by single-qubit interferometry. *Phys. Rev. Lett.* **110**, 230601 (2013).

[10] Dzhioev, A. A. & Kosov, D. S. Super-fermion representation of quantum kinetic equations for the electron transport problem. *J. Chem. Phys.* **134**, 044121 (2011).
